# The Critical Importance of Molecular Biomarkers and Imaging in the Study of Electrohypersensitivity. A Scientific Consensus International Report

**DOI:** 10.3390/ijms22147321

**Published:** 2021-07-07

**Authors:** Dominique Belpomme, George L. Carlo, Philippe Irigaray, David O. Carpenter, Lennart Hardell, Michael Kundi, Igor Belyaev, Magda Havas, Franz Adlkofer, Gunnar Heuser, Anthony B. Miller, Daniela Caccamo, Chiara De Luca, Lebrecht von Klitzing, Martin L. Pall, Priyanka Bandara, Yael Stein, Cindy Sage, Morando Soffritti, Devra Davis, Joel M. Moskowitz, S. M. J. Mortazavi, Martha R. Herbert, Hanns Moshammer, Gerard Ledoigt, Robert Turner, Anthony Tweedale, Pilar Muñoz-Calero, Iris Udasin, Tarmo Koppel, Ernesto Burgio, André Vander Vorst

**Affiliations:** 1 Association for Research Against Cancer (ARTAC), 57/59 rue de la Convention, 75015 Paris, France; philippei.artac@gmail.com; 2 European Cancer and Environment Research Institute (ECERI), 1000 Brussels, Belgium; dcarpenter@albany.edu (D.O.C.); lennart_hardell@hotmail.com (L.H.); igor.beliaev@savba.sk (I.B.); drmagdahavas@gmail.com (M.H.); gerard.ledoigt@wanadoo.fr (G.L.); erburg@libero.it (E.B.); andre.vandervorst@uclouvain.be (A.V.V.); 3 The Science and Public Policy Institute, Washington, DC 20006, USA; glac44@aol.com; 4 Institute for Health and the Environment, University at Albany, Albany, NY 12222, USA; 5 Child Health Research Centre, Faculty of Medicine, The University of Queensland, South Brisbane, QLD 4101, Australia; 6 The Environment and Cancer Research Foundation, SE-702 17 Örebro, Sweden; 7 Center for Public Health, Department of Environmental Health, Medical University of Vienna, 1090 Vienna, Austria; michael.kundi@meduniwien.ac.at (M.K.); hanns.moshammer@meduniwien.ac.at (H.M.); 8 Biomedical Research Center, Slovak Academy of Science, 845 05 Bratislava, Slovakia; 9 Trent School of the Environment, Trent University, 1600 West Bank Drive, Peterborough, ON K9J 0G2, Canada; 10 Verum-Foundation for Behaviour and Environment c/o Regus Center Josephspitalstrasse 15/IV, 80331 München, Germany; franz.adlkofer@googlemail.com; 11 Formerly UCLA Medical Center, Department of Medicine, P.O. Box 5066, El Dorado Hills, Los Angeles, CA 95762, USA; toxguns@netscape.net; 12 Dalla Lana School of Public Health, University of Toronto, Toronto, ON M5S, Canada; ab.miller@sympatico.ca; 13 Department of Biomedical Sciences, Dental Sciences and Morpho Functional Imaging, Polyclinic Hospital University, 98122 Messina, Italy; daniela.caccamo@unime.it; 14 Department of Registration & Quality Management, Medical & Regulatory Affairs Manager, MEDENA AG, 8910 Affoltern am Albis, Switzerland; chiara.deluca@medena.ch; 15 Medical Physicist, Institute of Environmental and Medical Physic, D-36466 Wiesenthal, Germany; vonklitzing@umweltphysik.com; 16 School of Molecular Biosciences, Washington State University, Pullman, WA 99164, USA; martin_pall@wsu.edu; 17 Oceania Radiofrequency Scientific Advisory Association (ORSAA), P.O. Box 152, Scarborough, QLD 4020, Australia; pri.bandara@orsaa.org; 18 Faculty of Medicine, Hebrew University of Jerusalem, Jerusalem 91905, Israel; yael.stein1@mail.huji.ac.il; 19 Hadassah Medical Center, Department of Anesthesiology, Critical Care and Pain Medicine, Jerusalem 91905, Israel; 20 Sage Associates, Montecito, Santa Barbara, CA 93108, USA; sage@silcom.com; 21 Istituto Ramazzini, via Libia 13/A, 40138 Bologna, Italy; soffrittim@ramazzini.it; 22 Collegium Ramazzini, Castello di Bentivoglio, via Saliceto, 3, 40010 Bentivoglio, Italy; 23 Environmental Health Trust, P.O. Box 58, Teton Village, WY 83025, USA; ddavis@ehtrust.org; 24 School of Public Health, University of California, Berkeley, CA 94720, USA; jmm@berkeley.edu; 25 Medical Physics and Medical Engineering Department, School of Medicine, Shiraz University of Medical Sciences, Shiraz P.O. Box 71348-14336, Iran; mortazavismj@gmail.com; 26 Ionizing and Non-ionizing Radiation Protection Research Center (INIRPRC), Shiraz University of Medical Sciences, Shiraz P.O. Box 71348-14336, Iran; 27 A.A. Martinos Centre for Biomedical Imaging, Department of Neurology, MGH, Harvard Medical School, MGH/MIT/Harvard 149 Thirteenth Street, Charlestown, MA 02129, USA; drherbert@bodybrainresilience.com; 28 Department of Hygiene, Karakalpak Medical University, Nukus 230100, Uzbekistan; 29 Department of Pediatrics, Medical University of South Carolina, Charleston, SC 29425, USA; robertturner@networkneurology.com; 30 Clinical Pediatrics and Neurology, School of Medicine, University of South Carolina, Columbia, SC 29209, USA; 31 Rebutting Industry Science with Knowledge (R.I.S.K.) Consultancy, Blv. Edmond Machtens 101/34, B-1080 Brussels, Belgium; anthonytweedale@telenet.be; 32 Foundation Alborada, Finca el Olivar, Carretera M-600, Km. 32,400, 28690 Brunete, Spain; pilar@fundacion-alborada.org; 33 EOHSI Clinical Center, School of Public Health, Rutgers University, Piscataway, NJ 08854, USA; iu22@eohsi.rutgers.edu; 34 AI Institute, University of South Carolina, Columbia, SC 29208, USA; tarmo@koppel.ee; 35 European Microwave Association, Rue Louis de Geer 6, B-1348 Louvain-la-Neuve, Belgium

**Keywords:** electrohypersensitivity, provocation test, electromagnetic field, radiofrequency, extremely low frequency, diagnostic criteria, biomarkers, pathophysiological mechanism, imaging techniques

## Abstract

Clinical research aiming at objectively identifying and characterizing diseases via clinical observations and biological and radiological findings is a critical initial research step when establishing objective diagnostic criteria and treatments. Failure to first define such diagnostic criteria may lead research on pathogenesis and etiology to serious confounding biases and erroneous medical interpretations. This is particularly the case for electrohypersensitivity (EHS) and more particularly for the so-called “provocation tests”, which do not investigate the causal origin of EHS but rather the EHS-associated particular environmental intolerance state with hypersensitivity to man-made electromagnetic fields (EMF). However, because those tests depend on multiple EMF-associated physical and biological parameters and have been conducted in patients without having first defined EHS objectively and/or endpoints adequately, they cannot presently be considered to be valid pathogenesis research methodologies. Consequently, the negative results obtained by these tests do not preclude a role of EMF exposure as a symptomatic trigger in EHS patients. Moreover, there is no proof that EHS symptoms or EHS itself are caused by psychosomatic or nocebo effects. This international consensus report pleads for the acknowledgement of EHS as a distinct neuropathological disorder and for its inclusion in the WHO International Classification of Diseases.

## 1. Introduction

Two of us—referred below as members of a “French research team”—recently published scientific evidence that electrohypersensitivity (EHS) is a distinct and objectively characterized neurologic pathological disorder, which can be diagnosed and treated using molecular biomarkers and imaging techniques [1]. However, it was later argued that the molecular biomarkers used in the study were not correlated with electromagnetic field (EMF) exposure, meaning this study did not prove EHS is caused by EMF [2].

Because the aim of that clinical study was to identify and characterize EHS as a distinct pathological disorder and not to prove that EMF can cause EHS-associated symptoms or EHS itself, we here respond to any scientist who may question the critical role of using molecular biomarkers and imaging techniques to objectively identify and characterize environmental diseases such as EHS and multiple chemical sensitivity (MCS). In addition, we emphasize that clinical research is the critical initial research step allowing disease characterization and definition. Without first having defined objective diagnostic criteria, studies looking for pathogenesis or etiology are of limited value, because they may result in confounding biases and medical misinterpretations. Indeed, clinical research allows one to diagnose diseases, understand pathophysiologic molecular mechanisms, and provide information on how to treat patients.

In medicine there are two different research paradigms: one is clinical research, mainly consisting of descriptive studies to identify and characterize diseases; and the other is so-called “public health research”, mainly consisting of comparative epidemiological studies in addition to experimental toxico-biologic data, to assess the causes and consequences of diseases at population level.

In this scientific consensus report, first, we emphasize how different these two complementary medical research domains are, and second, we explain why confusing the objective of these two different research approaches leads to scientifically unfounded claims, as is presently the case for environmental sensitivity illnesses, more particularly for EHS.

## 2. What Is Clinical Research

Since its foundation by Hippocrates, clinical research remains the basis of medicine [3,4,5]. It consists first in describing diseases, without necessarily considering causality. In ancient times, the approach was essentially based on clinical observation. However as soon as biology—more precisely biochemistry—evolved rapidly in the 19th century, biological tests were created and used to supplement disease symptomatic description and to define diseases more objectively. More precisely, since 1998 biomarkers used in clinical practice and research have been defined by the US National Institutes of Health (NIH) Biomarkers Definition Working Group [6], and more recently by the Food and Drug Administration (FDA)-NIH Biomarker Working Group BEST [7], as measured and objectively evaluated indicators that can be routinely and repeatedly used to objectively characterize diseases and their development. Moreover, the need to ensure sensitivity, specificity and reproducibility of the biological tests used to identify such indicators has been emphasized [8]. This is what the French team has done in previous studies by choosing adequate biological tests specifically dedicated for clinical research and qualified by reference laboratories for their sensitivity, specificity and reproducibility [9]. The results achieved by the clinical use of biomarkers may correlate with symptoms and disease types but usually not with initial causes. Thus, practically these tests cannot be used to identify the causal origin of diseases but only to establish disease nosological identification and classification. An example is cancer, for which different peripheral blood and/or urine molecular biomarkers are routinely used to diagnose various cancer types, evaluate treatment effectiveness and allow appropriate decision-making during the follow-up of patients. However, it is well known that cancer is a multifactorial disease associated with different risk factors; more specifically, it is caused by different physical, chemical and/or microbial environmental agents [10,11,12,13]. Thus, biomarkers as defined above cannot be used to assess the causal origin of cancer because they generally do not testify to causality. This consideration also applies to many other diseases—for example, diabetes type 2, obesity, cardiac disorders, Alzheimer’s disease and other types of degenerative neuropathies—for which molecular biomarkers and imaging information may reflect disease biological characteristics and nosological identification [14,15,16] but not specifically to initial causality, as the etiology of many of these diseases is multifactorial and is still not precisely determined [17].

As to etiology, it is important to distinguish it from pathogenesis. Indeed, etiology—with the exception of hereditary genetic diseases—refers primarily to environmental causes occurring in genetically susceptible hosts, whereas pathogenesis refers to molecular processes and pathophysiologic mechanisms involved from disease origin to final disease bioclinical presentation. In EHS, which is characterized and defined by the existence of hypersensitivity to man-made EMF (see below), this distinction is critical, because as for other diseases, biomarkers may reflect intra-corporeal pathophysiologic mechanisms but do not specifically reflect initial environmental causes.

Another feature that distinguishes clinical research from public health research is that clinical research most often uses descriptive studies for identifying and characterizing diseases clinically and biologically, whereas public health research mainly uses comparative epidemiological studies with the aim of determining disease etiology and risk factors involved at the population level.

## 3. What Is Public Health Research, and How Does It Differ from Clinical Research

The objective of public health research differs from that of clinical research as defined above. It consists of assessing the causes and consequences of diseases on populations located in defined regional or subregional areas, countries or even at a worldwide level to establish demographic, living conditions and more specifically environmental health risk factors so as to focus on disease causal origin [18]. Its main investigative methods are comparative descriptive epidemiology, multi-parametric studies and dose–response analyses. However, that is not enough, because epidemiology *stricto sensu* cannot prove causality but only the existence of an association between a possible causal factor and a health effect, no matter its retrospective or prospective analysis method. Thus, for causality assessment, even when a dose–response effect can be established in epidemiological studies, epidemiology needs to be complemented by the support of comprehensive in vitro and/or in vivo experimental toxicologic and biologic data, as proposed by the nine Bradford Hill criteria for demonstrating causality [19].

A good example showing how etiology research proceeds—and more precisely, what the contributing role of associated laboratory findings is—is cancer. Several epidemiological studies including those for radiofrequency EMF radiation (RFR) [20,21,22,23,24,25,26] and extremely low frequency EMF (ELF EMF) [23,27,28,29] have shown an associative link between EMF exposure and cancer or leukemia; thus, the International Agency for Research on Cancer (IARC) classified ELF EMF in 2001 [30] and RFR in 2011 [31] as possibly carcinogenic to humans (group 2B). Although the association found in these epidemiological studies could allow a possible causal interpretation, in fact, chance, biases and confounding factors could not yet be ruled out with sufficient certainty. Thus, these epidemiological data could not definitively establish the carcinogenic causal effect of man-made EMFs [32]. Additionally, it was considered at that time that there was insufficient comprehensive toxico-biologic data. However, this seems to be no longer the case, since two recent large independent in vivo studies, simulating human EMF exposure as closely as possible, have documented an increase in cancer in laboratory animals submitted to RFR or ELF EMF exposure. In the first of these studies, performed by the US National Toxicology Program (NTP), rats were exposed to whole-body RFR at 900 MHz and mice to whole-body RFR at 1900 MHz (both types of RFR exposure being combined with ELF EMF mobile phone pulsations) [33,34], while in the second study, performed by the Italian Ramazzini Institute, rats were exposed from the prenatal life until natural death to 1.8 GHz Global System for Mobile communication (GSM) [35]. As to mobile phone pulsations, ELF EMF studies demonstrated that exposure to Sinusoidal-50Hz magnetic field from prenatal life until natural death significantly enhances the carcinogenic effects of well-known human carcinogens [36,37]. Consequently, in addition to the results obtained from epidemiological studies, those two new independent laboratory findings and many independent earlier studies conducted in animals—including a US Air Force study in 1992 [38] and others showing RFR-related DNA genetic damage and oxidative stress induction at low radiation intensity level [39,40,41]—all led several scientists to consider that the evidence is sufficiently robust to re-classify RFR and ELF EMF as probably carcinogenic to humans, group 2A, or even as carcinogenic, group 1 [42,43,44].

Beyond the criteria of carcinogenic risk assessed by IARC, EMF exposure causality must also satisfy the criteria proposed by the WHO: (a) “the existence of biological effects and health hazards can only be established when research results are replicated in independent laboratories or supported by related studies”; (b) “there is agreement with accepted scientific principles”; (c) “the underlying mechanism is understood”; (d) and finally, “a dose-response relationship can be established” [45]. Consequently, before setting up and conducting research investigations on causality, pathologic disorders should have been previously clearly defined and objectively characterized; therefore, etiology and pathogenesis research as defined above can be investigated under independent and reproducible scientific conditions.

However, this is still not the case for EHS because, unfortunately, researchers initiated causality research before EHS was clearly identified, fully described and objectively recognized as a distinct pathological disorder. This is indeed an illogical methodological approach, leading to potentially confounding biases and confusing interpretations. As for the use of sham versus EMF exposure provocation tests in EHS self-reported patients, there remains persistent confusion between etiology and pathogenesis research. As previously emphasized, a distinction should be made between the cause of clinical symptom development in EMF-related hypersensitive patients, i.e., after EHS has already occurred (the pathogenesis), and the environmental causal origin of EHS itself (its etiology). Thus, provocation tests used in EHS patients cannot investigate etiology but only hypersensitivity-related symptoms. Moreover, in both the etiology and pathogenesis research domains, objective disease diagnostic criteria must be established before any specific investigation can be set up for obtaining valid conclusions. This is unfortunately not the case for most provocation studies so far achieved in EHS patients, which have been often carried out in a limited number of selected patients without using EHS-related objective inclusion criteria and suitable assessment endpoints (see below). This demonstrates why EHS first should have been objectively defined as a distinct pathological disorder thanks to the use of critical and rigorous methods of clinical research, as had been done previously and had been published by several research groups in different peer-reviewed scientific articles [1,9,46,47,48,49,50], rather than attempting to search for EMF-related causality before EHS was objectively defined.

## 4. The Case of Electrohypersensitivity

After its seminal identification and the creation of the concept of “electromagnetic sensitivity” by William J Rea in 1991 [51], EHS has been recognized as an emerging pathological condition, following many international consensus meetings that attempted to define it symptomatically. EHS was acknowledged by the WHO in 2005 as a disabling condition with non-specific symptoms and with no medical diagnosis and no evidence of causal origin [52]. Following the WHO-sponsored scientific consensus meeting in 2004 in Prague, this morbid condition was called “idiopathic environmental intolerance” (IEI) attributed to electromagnetic fields (IEI-EMF) [53], which suggests environmental intolerance possibly caused by EMF-related environmental exposure. However, because this pathological condition was essentially described symptomatically (similar to the previously described microwave syndrome [54]), several research groups including the French research team [1,9,48,49,50] and others [46,47,50] have attempted to search for molecular biomarkers and radiological criteria to objectively identify and characterize EHS.

The French team showed for the first time that EHS is associated with MCS in about 30% of cases and that overall EHS and MCS are associated with a similar clinical picture and biological signature. Thus, there may be two different etiopathogenic causal mechanisms of a common pathological disorder [9]. Moreover, on the basis of physical examination of the patients, this team showed that not all symptoms are subjective, since many cases’ cutaneous lesions, and possibly neurological physical abnormalities, could be objectively detected making EHS a true neurological, pathological disorder [1]. This last finding has also been observed by others who have published case reports showing the role of RFR exposure in inducing neurologic change [55,56] and in a single clinical study in which EHS has been ascribed as a neurological syndrome [57]. In addition, it has been shown that in EHS patients RFR exposure increases plasma glucose levels [58,59] and affects heart rate variability [59] and that in multiple sclerosis-bearing patients [60] RFR exposure can worsen symptoms, meaning that RFR can induce objective, bioclinical alterations in humans. These different objective findings are contrary to what has been published in the scientific literature using simple questionnaires and/or interviews tending to show that EHS is associated with subjective symptoms [61,62,63,64,65]. Indeed, contrary to these unfounded claims, not all symptoms are subjective; most symptoms are neurologic and usually not psychiatric [1,66]. The French team also showed that the biomarkers detected in the peripheral blood and urine of EHS patients reflect low-grade inflammation and oxidative/nitrosative stress [1,9,48], as was also shown in an Italian peer-reviewed publication [46]. These alterations are indeed non-specific biological features since they are also found in common diseases such as cancer, diabetes, obesity, Alzheimer’s disease and other suspected environment-related pathological disorders [67]. Nevertheless, they strongly testify to the somatic non-psychological signature of EHS, as is the case for many nosologically recognized diseases included in the International Classification of Diseases (ICD). Moreover, using different imaging techniques, including ultrasonic cerebral tomosphygmography (UCTS) [49,50], transcranial Doppler of the middle cerebral arteries [1] and functional magnetic resonance imaging (fMRI) [47], it was shown that EHS is associated with brain neurovascular dysfunction, even with potentially neuronal lesions, primarily involving the temporal lobes—most particularly the limbic system as well as other parts of the brain [47,49,50]. Clearly these data, which have been recently analyzed in a review of EHS-associated pathophysiological mechanisms [68], show the path to follow if we want to learn more about EHS and MCS, understand their pathophysiological molecular signature, and—most of all—if we want to determine efficient treatment and prevention methods based on rigorous scientific data.

## 5. Why Recent Scientifically Unfounded Claims Are Deeply Confusing

From a peer review scientific perspective, the criticisms made recently in a blog [2]—that the French team’s scientific publications were associated with inclusion problems and so studied non-EHS patients instead of EHS patients and should have used so-called “psychological” provocation tests instead of cohort analysis to prove EHS is caused by EMF exposure—are not justified. Although any blog has per se no scientific value, we wish to respond precisely to these unfounded criticisms because the scientific data obtained by the French team and other researchers may encourage more effective research on EHS.

### 5.1. Database Selection of Patients

Contrary to what was incorrectly assumed, patients included in the EHS patient database prospectively built up since 2009 by the French team were not selected on their EHS self-diagnosis, but, as previously reported [9,48], they were selected based on six major clinical criteria: (a) absence of known pathology accounting for the observed clinical symptoms; (b) reproducibility of symptom occurrence under the influence of suspected environmental sources, whatever they are; (c) regression or disappearance of symptoms in the case of presumed environmental source avoidance; (d) chronic evolution of symptoms occurrence; (e) symptomatic picture similar to that described in published peer-reviewed scientific literature for EHS and MCS; (f) no preexisting associated pathology such as atherosclerosis, diabetes, cancer and/or neurodegenerative or psychiatric diseases that would have rendered the interpretation of clinical and biological data difficult.

Note that most of these criteria were close to those used in defining MCS in a 1999 international consensus meeting [69] and in its subsequent proposed revision [70], and were adapted to EHS using similar basic criteria, in coherence with those reported by the WHO [45]. We agree that criteria (b) and (c) are dependent on a patient’s subjective feelings. However, as documented in different previously published papers [9,48,66], the self-reported diagnosis of EHS claimed by the patients was not taken into account; instead, all available anamneses and clinical examination-related pre-inclusion data were carefully analyzed before the case was recorded in the database. When the French team started recording these patients, objective biological and/or radiological diagnostic criteria for EHS were not yet established. In fact, there was no other means to define such a population sample, and thus no other available criteria to include patients in the database. It should also be considered that these clinical inclusion criteria were more complete and precise than those commonly used in so-called provocation studies (see below); however, if the inclusion criteria and endpoints are precisely defined and respected, the use of cohort studies in clinical research is fully justified. Unlike case-control studies, prospective cohort studies are not affected by recall biases. Finally—taking into account the above-reported clinical inclusion criteria used in previous studies by the French team—the suspicion that this team studied non-EHS and/or non-MCS patients instead of true EHS and/or true MCS patients can be dismissed since these clinical inclusion criteria and the cohort method analysis used were those recommended by the WHO [45].

### 5.2. Biomarker Expression and EMF Exposure

As mentioned above, biomarkers used in clinical practice and research should be distinguished from any laboratory findings, as they must be disease indicators routinely used and repeatedly measurable by biological tests complying with robust methodology. A serious mistake is to confuse the objective of clinical research using biomarkers and imaging techniques with that of causality research. As we have repeatedly emphasized, the objective of clinical research investigating EHS is not to assess the causal role of EMF and/or chemicals (or other possible contributing risk factors) or their presumed effects as symptomatic triggers but rather to define and characterize EHS itself clinically and biologically. Thus, biomarker analyses were never meant to be correlated with EMF and/or chemical exposure but were instead meant to objectively define EHS and to characterize its pathophysiological molecular mechanisms. These have now been shown to primarily involve low-grade inflammation, oxidative/nitrosative stress and, consequently, blood–brain barrier opening [1,9,49].

**Figure 1:**
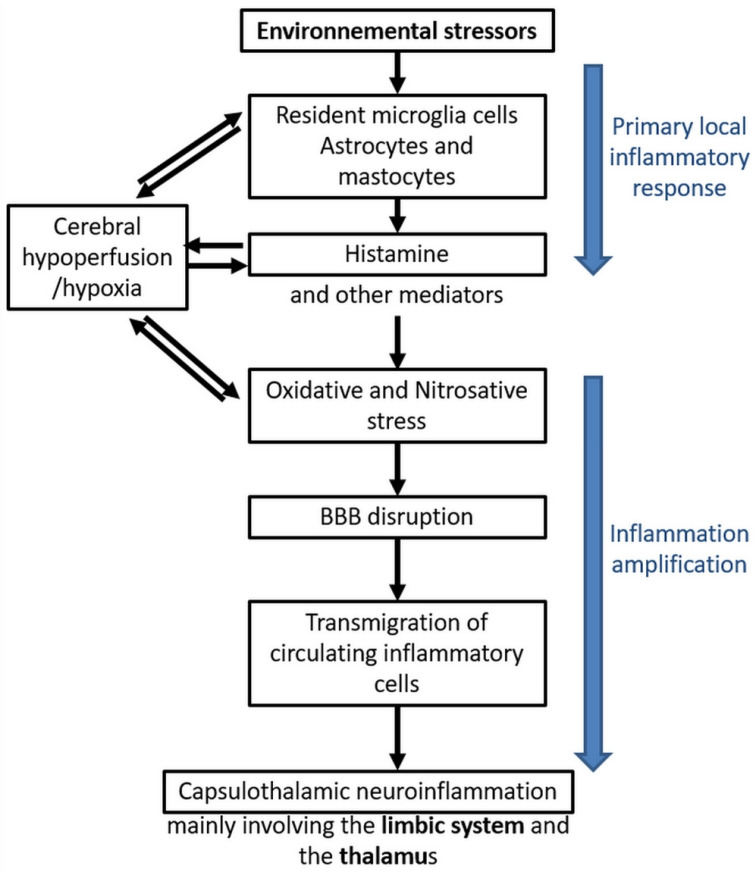
EHS/MCS physiopathological model based on low-grade neuroinflammation and oxidative/nitrosative stress-induced blood–brain barrier disruption, according to Reference [9].

It is important to recall that these different molecular abnormalities have been proven to be caused by EMF and/or chemicals in different independent in vitro and in vivo laboratory experimental studies [71,72,73] and that children have been shown to be more vulnerable to EMF exposure than adults [74], a finding that has led to the hypothesis that EMF could be involved as a causal agent in autism [75,76]. Among the experimental studies, the fact that man-made (artificial) waves physically differ from those of natural origin is of critical importance [77] since the pulsed and polarized physical characteristics of artificial waves emitted by wireless technology may account for their toxico-biological effects. This consequently may serve as a guide for future research [78]. In addition, it is now believed that man-made EMF may act on cells through coherent biological oscillations [79,80]. In particular, there may be some calcium ion channel activation that produces biological and health effects at non-thermal levels, a model that was developed for the first time by Dimitris J. Panagopoulos et al. [81] and more recently further documented [82,83]. In addition, several earlier experiments tried to study in particular the effects of low intensity millimeter waves (MMW) exposure in animal and human peripheral nerve tissues, but these studies have failed to reproduce EHS-associated neurologic effects, to the contrary providing some unexpected therapeutic beneficial effects [84]. These diverse independent but overall convergent experimental studies are the basic component of clinical research [58,59,70,77,80,85,86,87,88,89,90,91,92,93,94], which were analyzed during a consensus meeting on EHS held in Brussels in 2015 [95] and published in a peer-reviewed special issue [96]. Contrary to the scientifically unfounded statement of the International Commission on Non-Ionizing Radiation Protection (ICNIRP), a non-governmental German organization with supposed close links with the industry [97], the physical and biological data obtained from these experimental studies strongly suggest that non-thermal (or microthermal) health effects can be caused in animals as well as in humans by low intensity non-ionizing radiation [66]. Therefore, taking into consideration the pathophysiological data that have been so far obtained, a working hypothesis was proposed suggesting that EHS and MCS are pathological disorders of the brain, as has been suggested by imaging techniques, and that under the general term “environmental stressors”, environmental EMFs and/or chemicals may be causally involved in their pathogenesis, as suggested by in vitro experimental and clinical data [1,9,48] (Figure 1). This remains, however, to be confirmed, as (in association with this central nervous system hypothesis) in other exposure circumstances, a direct effect of EMFs in peripheral nerve tissues may occur (see below). Nevertheless, whatever the validity of this cerebral hypothesis, we conclude that by using molecular biomarkers and imaging techniques EHS and MCS can be objectively diagnosed and that patients can be treated on the basis of the presumed new pathophysiological mechanisms so far individualized, considering however that the overall sensitivity and specificity of the method used to diagnose these pathological disorders should be more precisely defined in the near future.

Figure 1 summarizes the different steps of the pathophysiological model the French team has so far been able to construct from the presently available published data. On the basis of low-grade inflammation, oxidative/nitrosative stress and blood–brain barrier opening processes, this model attempts to account for the mechanisms through which pathophysiological effects could take place in the brain of EHS and/or MCS patients and how EHS and/or MCS pathogenesis may consequently occur. The different model steps were developed by taking into account many available scientific data provided in the French team’s previously published articles [1,9]—in particular, for microglia cells, astrocytes and mastocytes [98,99,100,101], cerebral hypoxia [102,103,104,105,106,107,108], histamine [102,109,110,111,112], oxidative/nitrosative stress [48,113,114,115,116,117], blood–brain barrier disruption [103,104,105,106,107,108,112,118], and for the transmigration of inflammatory cells from the peripheral blood [119,120].

A further attempt to discredit the peer-reviewed work published by the French team was the allegation that the methodology used to investigate patients was not rigorous. For example, it was alleged that in the first study of this research group [9], 489 out of 1296 patients were artificially “excluded” from the study for unknown reasons. This is not correct. As the paper indicates, the 489 patients were simply excluded because they had not yet been fully analyzed for clinical findings, biomarkers and imaging technique. Another unfounded claim was that since many of the investigated patients from this cohort study came from different geographic areas, such as Europe, USA, Canada, Australia, etc., it was alleged that a reliable quality of sample collection could not have been assured because the measurement of markers would have been done in the geographical area where patients lived. However, the paper reported that all investigated patients in this cohort came to visit the French team in Paris and that all patients’ biological tests were performed in a unique, experienced biological laboratory in Paris to investigate patients under similar conditions through the use of standardized “good laboratory practice” technical conditions.

### 5.3. “Psychological” Provocation Tests

Limiting the study of EHS to the use of “psychological” provocation tests and to biochemical tests looking for “physiological” markers is a very restrictive and limited research objective [2], as it does not include clinical research, epidemiology, toxicology, and biology studies, and other domains of scientific investigations, and it fails the different WHO recommendations for causality assessment [45]. In addition, the term “psychological” is not a good label because sham versus EMF exposure tests should not explore the psychological behavior of EHS patients. Rather they should attempt to test pathogenesis, i.e., the putative role of EMF exposure in inducing EMF-related intolerance in patients presumably considered as hypersensitive to EMF [121,122,123,124,125,126]. Consequently, as mentioned above, when based on non-objective assessment, these tests generally cannot provide scientifically credible data for the role of EMF exposure in inducing EMF-related effects [122]. This may explain why many studies using provocation tests in EHS patients have reported negative findings (i.e., on the basis of subjective endpoints, patients fail to distinguish sham from EMF exposure). Moreover, we should also consider that in some cases these “negative” results may be due to the fact that not all sources of electromagnetic pollution were eliminated from the tested environment, so a person who is sensitive to ELF EMF or to intermediate frequencies (e.g., “dirty electricity”) could be reactive to “uncontrolled” frequencies rather than to the RFR being tested.

These negative findings have unfortunately reinforced the speculation made by psychologists who performed earlier studies funded by the UK telecom industry [121] that EHS is a psychological not somatic disease [121,122,123,124]. However, contrary to such a hypothesis, this does not mean that EHS patients can tolerate EMF exposure, because—as depicted in Table 1—there are many methodological inclusion and assessment problems and medical interpretation pitfalls associated with the use of these tests. Indeed, we must recall that in order to bring about a scientifically credible assessment (i) any provocation study should use objective inclusion criteria based on initial somatic characterization of EHS, (ii) methodological procedures should be based on rigorous and suitable objective parameters as close as possible to the patient’s real exposure conditions, and (iii) a clear definition of the type of objective endpoint should be considered [57]. Indeed, subjective endpoints should be limited and even avoided (see below) in any provocation studies because of the possible lag phase between exposure to EMF and symptoms occurrence and because once symptoms have occurred it may be difficult for the patients to distinguish real exposure from sham exposure. (see Table 1).

Clearly, not all the three steps indicated in the above reported procedure were followed in the majority of provocation studies, meaning that the present dogmatic psychological interpretation of negative results is scientifically irrelevant. In addition, besides dependencies on carrier frequency, modulation, genotype, physiological traits and the presence of radical scavengers and antioxidants—all reported by many research groups—emerging data suggest dependencies of the RFR effects on polarization, intermittence and coherence time of exposure, as well as stray electromagnetic fields during exposure [78]. Most of these parameters were not considered in the provocation studies, rendering most of those studies inconclusive.

In fact, not all provocation studies in EHS patients have resulted in negative findings, meaning that EMF exposure including RFR and ELF EMF exposure could be potential triggers of biological and clinical effects in such patients. For example, in their 2011 systematic review of 122 provocation studies, Rubin et al. [124] arbitrarily selected 29 single or double-blind studies assessed as having respected the review’s selection protocol (i.e., having used a correct methodology), and considered 5 of these studies [51,131,132,133,134] as being reliably associated with positive effects following EMF versus sham exposure—meaning that in so called IEI-EMF patients, EMF could induce various objective physiological alterations, such as heart rate and/or blood pressure variability, altered papillary light reflex, reduced visual alteration and perception, altered electroencephalogram (EEG) during sleep and skin conductance modifications, etc. In comparison with sham exposure, symptomatic intolerance induction has also been reported, in a single EHS case double-blind procedure, to be caused by off–on or on–off field transition rather than the presence of EMF exposure itself, while the EHS patient had no perception of this exposure. As stated by the authors, this means that the statistically reliable somatic reactions to subliminal EMF exposure were obtained under conditions that reasonably excluded the causative effect of any psychological process [56].

Such positive data obtained by provocation tests have also been independently shown in two earlier different EHS case reports [56,57] and more recently in two studies showing in EHS patients the objective effect of pulsed microwave radiation on heart rate variability in a double-blind provocation study [58] and more generally the effects of RFR on the blood, the heart and the autonomic nervous system [59]. Similar objective endpoints were also provided independently by two German scientists—Andreas Tuengler and Lebrecht von Klitzing—who considered that heart rate variability, microcirculation (capillary blood flow) and electric skin potentials [135] and electromyogram (EMG) recording [136] were suitable non-invasive methods for use in provocation studies as an objective endpoint assessment.

By contrast, in so-called “psychological” provocation tests, the presumption is that subjects are conscious of their exposure, whether it is real or sham. This is an erroneous, non-objective presupposition because as indicated above there may be a significant delay from the exposure to the occurrence of any perceivable effect and because the subject may not be aware that the adverse effects are really taking place, whereas possibly biomarkers and the previously reported neurologic- and skin-based objective response (as measured by heart rate variability, microcirculation, electric skin potentials or altered EMG) indicate the occurrence of induced i.e., causal, effect [135,136,137].

Consequently, contrary to persistent unfounded claims of psychological etiology, these diverse independent findings strongly suggest a causal role of EMF in inducing bioclinical effects in EHS patients, as is the case for allergic patients for whom an increase in allergic response upon acute EMF exposure has also been observed [138,139]. This led us to seriously discuss the overall irrelevant EHS-associated inclusion criteria and the artificial selection made by Rubin et al. in his so-called review of provocation studies [124] (see below the Discussion section).

While the present medical state-of-art should avoid any psychological etiology and pathogenesis causal interpretation for EHS occurrence and symptomatic development, it remains nevertheless a first-order, fundamental open question. Could these tests support the concept of EHS patients’ hypersensitivity to EMF, i.e., that EHS patients are more sensitive to EMF than “healthy” subjects? A preamble to this important question is that it has been reported that healthy subjects would not show any change in heart rate variability, microcirculation and electric skin potentials under exposure to EMF in comparison with the unexposed state, whereas EHS patients exhibit typical changes in these three parameters overtime and thereafter [135]; this supposition has to be seriously discussed (see below). In fact, demonstrating hypersensitivity to EMF (i.e., the specific pathophysiological identification of EHS) should be the main objective of provocation tests in EHS patients. For such a goal EHS patients should be rigorously compared with normal healthy controls in case-control or randomized studies, and objective endpoints should be defined appropriately. Unfortunately, up to now such comparative studies have not been done. To our knowledge, hypersensitivity to EMF in EHS patients (i.e., the decrease in the EMF tolerance threshold) has still not been clearly demonstrated [124,140] because, contrary to previous reported supposition, several provocation studies that used healthy volunteers have also shown some biological effect induced by EMF exposure, including baro-reflex activity [141], increased glucose metabolism activity [142] and, more recently, heart rate variability depending on inspiration/expiration ratio [143].

Furthermore, there remains a second open question: Could provocation tests determine the attributable fraction to EMF exposure as a trigger of symptomatic and bioclinical intolerance in comparison with that of other putative triggers such as chemicals? Indeed, we now know that MCS is associated with EHS in about 30% of EHS patients [1,9], that in MCS, associated intolerance symptoms are triggered by chemical exposure [69] and that MCS is associated with clinical symptoms and biological abnormalities similar to those of EHS [1,9]; thus, chemicals could also be a symptomatic and bioclinical trigger in these patients. Moreover, a psychological stress may be associated with EMF and/or chemical exposure (see Table 1). Certainly, in future provocation tests in EHS patients, we should determine precisely the different attributable fractions of these potential triggers by using appropriate specific objective biomarkers in addition to symptomatic assessments.

Finally, this short review of provocation tests confirms that the effect of EMF on the organism depends on multiple physical and biological parameters, as has been shown experimentally in the laboratory [80]. Moreover, practically, there is no pure RFR, since in all technological applications RFR is combined with ELF EMF modulation and pulsing. Real complex signals are variable in intensity at each moment—the ELF components as well as the RF signal being bioactive [41]. In addition, as electric fields are physically associated with magnetic fields, it remains practically extremely difficult to clearly distinguish the effects of the ones from those of the others in a clinical and biological setting. This explains why, to reproduce reality, the use of provocation tests in humans is complex and why the results are difficult to interpret. Such critique should not however reject the use of these tests but should emphasize the need for well-designed provocation studies to complement the results obtained by clinical research using biomarkers and imaging techniques.

## 6. Discussion

As far as research on EHS is concerned, it appears today that there is a gap between scientists who practice medical research aimed at objectively defining EHS as a pathological disorder and those who believe that provocation tests, either in the laboratory or in humans, are best to support causality for EMF-related environmental intolerance and etiology. A possible explanation for the confusing objectives of these two different approaches is that—unlike other current pathological disorders such as cancer, diabetes, obesity, Alzheimer’s disease, cardiac disorders etc., for which their etiology and pathogenesis is not needed for their nosological identification—EHS is supposed to be a particular pathophysiological state of hypersensitivity to EMF and so it is presumed to be clinically and biologically associated with a causal intolerance to man-made EMF exposure [51,53,144,145]. This hypothetical EMF-related environmental causality was thus investigated by the use of provocation tests. Unfortunately, as emphasized above, since these provocation tests were mainly based on non-suitable objective biochemical/biophysical methods [146], most of them could not produce reliable results. A serious mistake made by Rubin et al. in their review of available provocation studies [124] is that the numerous studies they analyzed were in fact heterogeneous for inclusion and endpoint criteria and not carefully designed to effectively detect sensitivity responses to EMF in so-called IEI-EMF patients, whereas in the Rea initial study [51] and the two Havas studies [58,59], EHS patients might have been chosen according to prescreened initial conditions based on their consistent, measurable responses to EMFs. In contrast to such operative conditions, the different heterogeneous non-prescreened inclusion protocols used in the diverse provocation studies analyzed by Rubin et al. may explain why, in addition to a very high drastic selection of studies (see the above section about “psychological” provocation tests), there could be in many studies so far analyzed a possible hypothetical high dropout rate of hypersensitive patients who refused to be submitted to the studies or who had been lost to follow-up because they did not tolerate EMF exposure. Consequently, due to these two types of possible selection biases, it is proposed that the negative results obtained in a majority of provocation studies may have been artificially provided. Thus, it was erroneously speculated that the causes of EHS-associated symptoms and EHS itself could not be related to EMF exposure but to some nocebo effect, i.e., EHS is a psychological disease [124,147]. Given this hypothesis, some researchers initially reported that psychological factors could be involved in EHS [148]. However, when they collected sufficient data, they realized that EHS was not linked to psychological issues and thus rectified their previous inappropriate hypothesis [149]. Indeed, the so-called nocebo effect is, at best, a hypothesis that needs to be validated by specific suitable experimental studies. This has not occurred. To the contrary, it has been shown that the psychological problems associated with EHS were secondary to disease occurrence, not the cause [150]. Moreover, the molecular and radiological abnormalities that the French team and others have observed demonstrate that EHS is a somatic, not a psychological disease. Additionally—as indicated above—a further misinterpretation of provocation tests is that their objective was confused with research on etiology and was not restricted to a search for EMF exposure intolerance. Note, however, that MCS is believed not just to be a state of increased symptomatic sensitivity to multiple chemicals but also to be caused by environmental chemicals in genetically susceptible hosts [69].

These considerations, in addition to the lack of previous objective nosological identification of EHS, may explain why up to now the WHO has ascribed EHS as a morbid condition and not as a pathological disorder; thus, why EHS is still not included in the WHO ICD. A further consideration is that the ICD catalogue of the diverse nosologically identified diseases is separated from the description of their etiologies. Consequently, while EMF, including ELF EMF and RFR, have been independently acknowledged by the IARC as possibly carcinogenic [30,31], in the ICD-10, cancer is cited separately from EMF and other etiologies, as is the case for the other ICD-included diseases and pathological disorders. Thus, while ICD-10 chapter XX entitled “External causes of morbidity and mortality” includes non-ionizing radiation such as RFR under the code W90 as a potential cause of diseases [151], diseases potentially resulting from non-ionizing radiation are enumerated separately from their putative causal origins. Consequently, whatever the causal origin of EHS, there is presently no reason to believe that the WHO will accept EHS to be included in further ICDs as long as it is not recognized to be a distinct pathological disorder. Thus, clinical research attempting to objectively define and characterize EHS is of critical importance if we want EHS to be acknowledged as a distinct pathological disorder, and so, as proposed by the European Academy of Environmental Medicine (EUROPAEM) [152]) to be included in the future WHO ICDs.

As a further implication of all these issues, clinical research should be reinforced to more precisely define and elucidate still unexplained findings. Provocation tests should be repeated under more robust methodological conditions, including not only RFR exposure but also exposure to all forms of EMF, such as ELF and intermediate frequencies (“dirty electricity”)—but only after diagnostic inclusion criteria and assessment endpoints have been clearly and objectively defined. Lastly, in addition to in vitro and in vivo experimental laboratory findings using EMFs and chemicals as potential disease triggers, epidemiology should also be considered because it is a very efficient and relevant tool in public health research. In any case, it would be extremely inefficient and dangerous to restrict research on diseases to scientists not familiar with clinical research and medicine. In other words, it is through close collaboration between clinicians, epidemiologists and biologists—and also biophysicists and biochemists—that the truth will progressively emerge about EHS, which remains an intriguing nascent environmental pathology with worldwide high-risk public health implications [153] in our increasingly electromagnetically polluted world, due in particular to the widespread deployment of wireless technologies [154].

## Figures and Tables

**Table 1:** Some methodological defects that make provocation tests unsuitable for sham versus EMF exposure analysis in EHS-bearing patients.

1	Lack of precise inclusion criteria. No objective criteria based on molecular biomarkers and imaging techniques.	[62,125,127,128]
2	No clear consideration on medical anamnesis and degree of EHS severity.	[125,127]
3	No consideration for an association with MCS.	[9]
4	No consideration that EHS patients are intolerant to specific man-made waves frequencies.	[62,125,127,128]
5	Too short exposure duration.	[125,126]
6	Symptom recording made too early.	[125,127]
7	Endpoint criteria depending on subjective statements.	[62,122,123,124,125,126,127]
8	Possible EHS-associated psychological conditioning due to past suffering.	[129]
9	Possible abnormal EMF signal transmission in case of sham exposure.	[130]
